# The Role of the Membrane-Initiated Heat Shock Response in Cancer

**DOI:** 10.3389/fmolb.2016.00012

**Published:** 2016-04-27

**Authors:** Zohar Bromberg, Yoram Weiss

**Affiliations:** ^1^The Goldyne Savad Institute of Gene Therapy, Hadassah-Hebrew University School of MedicineJerusalem, Israel; ^2^Hadassah Medical OrganizationJerusalem, Israel

**Keywords:** heat shock protein, membrane receptors, cancer, TRPV cation channels, heat shock response

## Abstract

The heat shock response (HSR) is a cellular response to diverse environmental and physiological stressors resulting in the induction of genes encoding molecular chaperones, proteases, and other proteins that are essential for protection and recovery from cellular damage. Since different perturbations cause accumulation of misfolded proteins, cells frequently encounter fluctuations in the environment which alter proteostasis. Since tumor cells use their natural adaptive mechanism of coping with stress and misfolded proteins, in recent years, the proteostasis network became a promising target for anti-tumor therapy. The membrane is the first to be affected by heat shock and therefore may be the first one to sense heat shock. The membrane also connects between the extracellular and the intracellular signals. Hence, there is a “cross talk” between the HSR and the membranes since heat shock can induce changes in the fluidity of membranes, leading to membrane lipid remodeling that occurs in several diseases such as cancer. During the last decade, a new possible therapy has emerged in which an external molecule is used that could induce membrane lipid re-organization. Since at the moment there are very few substances that regulate the HSR effectively, an alternative way has been searched to modulate chaperone activities through the plasma membrane. Recently, we suggested that the use of the membrane Transient Receptor Potential Vanilloid-1 (TRPV1) modulators regulated the HSR in cancer cells. However, the primary targets of the signal transduction pathway are yet un-known. This review provides an overview of the current literature regarding the role of HSR in membrane remodeling in cancer since a deep understanding of the membrane biology in cancer and the membrane heat sensing pathway is essential to design novel efficient therapies.

## The heat shock response and cancer

The heat shock response (HSR) is a highly evolutionary conserved mechanism that is initiated by environmental and physiological stressors such as heat, oxidative stress, heavy metals, toxins, and bacterial infections, and is essential for the survival in a stressful environment (Akerfelt et al., 2010). The HSR is a cell-autonomous response and is thought to be induced by the presence of misfolded proteins. The cellular protein homeostasis (“proteostasis”) network was described recently in one of Morimoto's latest reviews as “a conserved cellular machinery to support all aspects of protein biogenesis, to adapt to a complex and changing environment, and to determine organismal lifespan.” Proteostasis is the process that integrates signals and regulates flux from protein synthesis, folding, transport, and degradation (Brandvold and Morimoto, 2015). The chaperone network assists in maintaining protein functionality and counteracts intrinsic and extrinsic forces that perturb protein folding. Hence, molecular chaperones maintain cellular, tissue, and organismal health (Brandvold and Morimoto, 2015).

Mutations, inflammation, neurodegenerative, and cancer diseases are associated with abnormal cytosolic and endoplasmic Reticulum (ER) proteostasis, hence, with changes in the expression of chaperones (Brandvold and Morimoto, 2015). Chaperones are a family of highly conserved proteins that recognize nascent polypeptides and folding intermediates and whose function is to guide proteins to their native state. Among them is the most highly evolutionary conserved, the Heat Shock Protein 70's (Hsp70) family (Bukau and Horwich, 1998; Dufey et al., 2015). Hsp70 binds to its substrates through hydrophobic residues in an ATP-dependent manner. This binding is accompanied by Co-chaperones such as: Hsp40 (Bukau and Horwich, 1998). Hsp70 also binds to mutant proteins that may accumulate in the cytoplasm and hence enabling the accumulation of cancer mutant proteins, such as p53 (Jäättelä et al., 1998; Voisine et al., 2010). Both Hsp70 and Hsp90 interact and regulate many transcription factors, signaling molecules, and kinases that are related to cancer, including NF-κB, p53, v-Src, Raf1 Akt, and steroid hormone receptors (Li et al., 2009). Hence, there is evidence that transformed cells express higher levels of Hsps compared to non-transformed cells (Jäättelä et al., 1998) and that Hsp70 plays a role in cancer development, since high levels of Hsp70 is also thought to increase the resistance of tumor cells to apoptosis (Jäättelä et al., 1998) or to chemotherapy (Mosser and Morimoto, 2004). Therefore, the anti-apoptotic role of the chaperones, mainly in cancer cells, may “contribute” to the increased resistance to chemo- and radiotherapy treatments.

Overexpression of Hsp70 can inhibit multiple pathways of cell death, including both intrinsic and extrinsic apoptosis (Murakami et al., 2015). Hsp70 is known for its ability to bind the pro-apoptotic Bcl-2 family member BAX, thus preventing its activation and translocation to the mitochondria (Murakami et al., 2015). Hsp70 also binds the death receptors DR4 and DR5 of the extrinsic apoptotic pathway. Hence, inhibition of cytosolic Hsp70 has emerged as a promising anti-cancer therapy (Murakami et al., 2015).

We have previously found that Hsp70 acts at multiple control points of the apoptotic pathways, including inhibition of several activation of caspases, prevention of cytochrome c release and regulation of the apoptosome by directly binding to Apaf-1 (Aschkenasy et al., 2011). Hence, since Hsp70 plays a general protective role against programmed cell death, increased expression of Hsp70 correlates with cancer progression and metastasis.

In tumor cells, ER stress also occurs by micro-environmental changes such as hypoxia, nutrient deprivation, acidosis, calcium depletion, glucose deprivation, and reactive oxygen species (ROS) production (Dufey et al., 2015). Although the ER chaperone network also suppresses tumorigenesis, [depending on the tumor context (van Vliet et al., 2015)], these stressful perturbations promote the Unfolding Protein Response (UPR) and the release of several factors that induce stress adaptation and cell survival, and might also increase resistance to chemotherapy (Dufey et al., 2015). For instance, ER chaperones such as the immunoglobulin heavy-chain Binding Protein (BiP) is one of the factors initiating tumor growth, becoming detectable on the cell surface of many cancer cells (Dufey et al., 2015). We know that BiP dissociated from protein kinase R -like ER kinase (PERK), inositol-requiring enzyme 1 (IRE1) and activating transcription factor 6 (ATF6). ER homeostasis is achieved by PERK translation as well as phosphorylation of the initiation factor 2α (eIF2α) in order to reduce influx into the ER (van Vliet et al., 2015). Interestingly, others have found that PERK also utilizes lipid kinase actvity, using diacylglycerol (DAG) as a substrate to generate phosphatidic acid (PA). This phosphorylation process was found to be correlated with the phosphorylation of Akt and activation of mTOR, thus, hinting that PERK has also a central role in cellular migration, metabolism, and cell growth. Due to the fact that PA is produced by PERK further points to its central role in membrane and actin remodeling and in several pro-survival pathways (van Vliet et al., 2015).

Recent works have also shown the role of PERK in ER calcium signaling, since it has been linked to calreticulin (Ca^2+^ dependent phosphatase, store operated calcium entry), as exemplified in a study done in colon cancer cells. It was previously shown that lower ER Ca^2+^ levels promote pre-apoptotic calreticulin exposure on the surface of cancer cells treated with chemotherapy (van Vliet et al., 2015).

## Regulation of the heat shock response—the classical pathway

The HSR is regulated at the transcriptional level by the activities of a family of heat shock transcription factors (HSFs). The mammalian HSF family consists of four members: HSF1, HSF2, HSF3, and HSF4 (Akerfelt et al., 2010). Different HSFs possess unique and overlapping functions, consisting of tissue specific patterns having various post-translational modifications (PTMs) (Akerfelt et al., 2010). Heat Shock Factors (HSFs) recognize and bind to heat shock elements (HSEs) present in the promoter of heat shock genes. Among the human HSF genes, HSF1 is the most essential for the HSR and is constitutively expressed in most tissues and cell types (Akerfelt et al., 2010). For many years, the consensus of the activation of the HSR was that under normal conditions of cell growth, HSF1 exists in a repressed state associated with Hsp90, Hsp70, and Hsp40 and distributed in a negatively regulated state as an inert monomer in the cytoplasm. Upon exposure to a variety of stresses such as heat shock, or oxidative stress, HSF1 is liberated, trimerizes, and accumulates in the nucleus (termed the “classical” pathway). HSF1 trimers bind with high affinity to the HSE. (Barral et al., 2004; Westerheide and Morimoto, 2005; Soto and Estrada, 2008; Voisine et al., 2010). However, there is also an interplay between HSFs, since HSF2 also binds to the promoter of HSP genes. HSF2 depends on HSF1 for its stress-related functions (Elsing et al., 2014). However, HSF2 modulates HSP gene expression, implying that HSF2 has a role in the regulation of HSR. It is known that HSF2 also plays a role in the developmental processes (Elsing et al., 2014). The expression of HSF2 in the testes is regulated by microRNA mir-18i (Björk et al., 2010). Mir-18 is also a member of the mir-17-92 oncomir-1 cluster and has been proposed to participate in tumor progression, as was demonstrated in a mouse model of colon cancer (Dews et al., 2006). HSF2 has an active role during mitosis and a decreased HSF2 expression during mitosis was shown to protect cells against preteotoxicity and apoptosis (Elsing et al., 2014). HSF2 deficient cells have also shown elevated levels of survival during acute heat stress, with fewer mitotic errors (Björk et al., 2010). Further, it was suggested that declining levels of HSF2 during mitosis in several cell lines is accompanied with induction of Hsp70 (Elsing et al., 2014). Hence, it is tempting to speculate that controlling the levels of HSF2 in cancer could affect Hsp70 expression. Others have demonstrated that HSF2 is a potent suppressor of tumor progression in prostate cancer by directing and regulating epithelial plasticity (Dews et al., 2006). An interesting fact is that HSF2 was found to regulate cell movement affecting actin cytoskeleton signaling (Björk et al., 2016).

Among the well-known substances that were found to affect the classical pathway activating or inhibiting the HSR are: Celastrol, Arylsulfanyl as well as Pyrazolones (Llauger et al., 2005). Arylsulfanyl, for instance, is a purine-scaffold class inhibitor (Llauger et al., 2005), and a series of 8-arylsulfanyl, -sulfoxyl, and –sulfonyl adenine members of the purine class were synthesized and evaluated as inhibitors of chaperones; they bind to Hsp90, (a highly abundant chaperone within the cells), with moderate affinity and initiate cellular effects that mimic 17-allyl-aminodesmethoxy- geldanamycin; (17AAG). This inhibitor, (17AAG) has entered clinical trials in cancer patients in the US and UK and has shown early advantage of therapeutic activity when administered alone or in combination with the chemotherapeutic docetaxel. However, 17AAG has a limited solubility and cumbersome formulation. It also exhibits dose dependent liver toxicity caused by the benzoquinone functionality (Chang et al., 2014).

Celastrol, which activates the HSR and elevates Hsp70 levels, inhibits the IKK-NF-κB cell signaling pathway, by inhibiting directly the IKKα and IKKβ and inactivating the Cdc37 and p23 proteins (Salminen et al., 2010).

Due to the biochemical and structural differences between Hsp90 and Hsp70, the targeting of Hsp70 has been less developed (Goloudina et al., 2012). Furthermore, inhibition of Hsp90 alone could also be associated with a compensatory increase in cytosolic Hsp70 levels leading to tumor resistance (Goloudina et al., 2012). Therefore, it is important to develop inhibitors of Hsp70 that will be used in conjunction with Hsp90 inhibitors.

Substances that inhibit Hsp90, such as 17-AAG, enhance the binding of GRP75 (a member of the Hsp70) to p53, resulting in the “holding” of p53 in the cytoplasm. However, inhibition of GRP75 using MKT-077 (rhodacyanine), an Hsp70 inhibitor, initiates disruption of the 17-AAG-induced GRP75-p53 complex. Released p53 translocates into the nucleus and triggers transcription of apoptosis-related genes (Guo et al., 2014), as was recently found in a liver cancer xenograft model (Guo et al., 2014). However, MKT-077 is rapidly metabolized, which limits its usability. Therefore, analogs of MKT-077 were designed with greater stability. One of such molecules is JG-98 (Li et al., 2013), which is about three-fold more active than MKT-077. JG-98, moderately destabilized Akt1 and Raf1, which are known to interact with each other and thus to stimulate proliferation (Zimmermann and Moelling, 1999).

Peptide inhibitors were also developed. One of them, ADD70, a domain of Apoptosis-inducing factor (AIF) interacts with the substrate binding domain (SBD) of Hsp70, with a promising anti cancer activity when transfected into tumor xenografts in mice (Murphy, 2013).

Gudkov discovered the 2-phenylethynesulfonamide (PES), a compound that inhibits the trafficking of p53 to the mitochondria, NF-kB activation and causes ribosome pausing as well as decreasing protein translation (Murphy, 2013). Hence, PES could serve as a potent Hsp70 inhibitor for the treatment of cancer.

PES was also proposed to bind to the SBD of heat-inducible Hsp70. PES also demonstrated moderate inhibitory effects on the chaperone action of the constitutive Hsc70 and the heat inducible Hsp70. However, it was suggested that PES may also interact with low affinity and un-specifically with the SBD of Hsp70 (Schlecht et al., 2013).

Others have used the structure of HSC70 –BAG-1 interaction to design analogs that bind to the ATP-binding pocket (Murphy, 2013), such as the ATP-analog VER-155008, which inhibits the proliferation of various cancer cell lines (Murphy, 2013).

However, since Hsp70 binds ATP about 300 times more tighter than Hsp90 and with the Hsp70's site being more exposed to solvent (Brandvold and Morimoto, 2015), we should question whether there is an alternative way for modulating chaperones activity. In several diseases such as cancer, the induction of the HSR is followed by the remodeling of membrane lipids (Gombos et al., 2011). So, is it possible to regulate the HSR in cancer through the use of a treatment that affects membrane lipid re-organization?

## The plasma membrane and cancer

The biological membrane acts as a selectively permeable barrier (Maxfield and Tabas, 2005), allowing the functions of organelles as well as the transmembrane electro-chemicals (Maxfield and Tabas, 2005). The biophysical properties of membranes such as fluidity is intimately linked to the composition and structural organization of membrane lipids (Maxfield and Tabas, 2005). pH, ionic strength, temperature, water concentration, or lateral pressure and the temperature modulate the membrane fluidity (Lladó et al., 2014). Today, we know that there is heterogeneity in lipid and protein populations in the plasma membrane. This complexity is present in lipid rafts, caveolae, and receptors/channel clusters, since many proteins such as channels, receptors, and enzymes, interact with lipids of the cell membranes (Lladó et al., 2014).

Changes in lipid composition or modification in the structure of the cell membranes have been described in a number of pathologies (Maxfield and Tabas, 2005). Several oncogenic processes are initiated through the plasma membrane, such as adhesion, proliferation and migration (Maxfield and Tabas, 2005). Protein-lipid interactions are modified following the disease state as well (Maxfield and Tabas, 2005). In cancer cells, there are changes in lipid composition that are related to the malignancy of the tumor; there is elevation of fatty acid biosynthesis in order to generate new membranes due to an increased proliferation ratio. With regard to the breast tissue it is known that the fatty acid and phospholipid profiles are also altered in the healthy tissue areas surrounding the tumor (Maxfield and Tabas, 2005).

One of the most important lipid regulators of the cell membrane is Cholesterol (Maxfield and Tabas, 2005). One important lipid pathway modified in cancer is the cholesterol metabolism, since esterification of cholesterol is linked to an elevation of the cell cycle progression and tumor growth. Changes in the composition of lipids can affect signal transduction and membrane trafficking; For instance, certain lipids, specifically phosphoinositides, organize signal-transduction processes located within the cells (Maxfield and Tabas, 2005).

Reduced cholesterol is associated with reduced actin-dependent protrusions and with reduced activation of the small GTPase Rac in cells (Maxfield and Tabas, 2005). The Rho family GTPases are central to various responses in the cell through the regulation of cytoskeleton organization, adhesion dynamics and membrane trafficking, cell cycle progression and survival (Moissoglu et al., 2014). Their dysregulation promotes several diseases as well as cancer (Moissoglu et al., 2014). For years, it has been suggested that Rac1 is associated with cholesterol-rich membranes, lipid rafts (Moissoglu et al., 2014). However, recently, it has been demonstrated that Rac is present in disordered domains and that Rac translocates mainly to the domain boundaries. It was also observed recently that Rac is selectively inactivated in non-raft regions (Moissoglu et al., 2014).

A high percentage of the approved drugs for treating cancer are directed toward the plasma membrane. Among them are drugs that are targeted to the tyrosine kinase receptors VEGF, HGFR, MAP kinase 1, and serine threonine-protein kinase B-raf (Ziegler et al., 2014). Ziegler et al. managed to screen target proteins that are present in the plasma membrane in breast cancer cell lines (Ziegler et al., 2014): They found the expression of the tyrosine kinase receptors that initiate signal transduction pathways critical to the development of normal cells. However, when de-regulated, they promote proliferation and metastasis (Ziegler et al., 2014). Other proteins found in the plasma membrane, such as adhesion molecules, are crucial for interactions with neighboring cells and the extracellular matrix. These molecules are highly expressed in the plasma membranes of several breast cancer cell lines. Among them are the cadherins, catenins, and integrins. Further, tumor progression is correlated with changes in the cytoskeletal protein profile and organization. The changes are thought to be initiated at the cellular surface level of the transformed cell that loses polarity and adhesion, with amorphous properties and migratory capabilities (Ziegler et al., 2014).

Some cancer cells have a lipogenic phenotype, in which they synthesize fatty acids for plasma membrane requirements. Lipogenesis is thought to be correlated with chemoresistance and protection from various insults, in order to increase survival under stressed conditions. Such insults could be mechanical stress and immune system attack, both known to cause plasma membrane injury and stressors that are faced by progressing tumors (Ziegler et al., 2014).

## The plasma membrane—the first shield combating the heat shock response

Over the last decade, a parallel paradigm has matured, placing the plasma membrane at the forefront and main effector of the HSR. The “membrane thermosensor” theory suggests that heat shock can induce changes in the fluidity of membranes, leading to remodeling of the membrane and re-organization of the membrane lipids. Such changes occur in cancer or neurodegenerative diseases, diet and during aging (Gombos et al., 2011), thus, influencing stress sensing cellular pathways that up-regulate heat shock proteins (Gungor et al., 2014). Since the membrane lipid phase structure is dependent on the thermal behavior of the membrane, the use of non-proteotoxic membrane fluidizers revealed that changes in the membrane surface are the first initiated events of the heat sensing pathway signaling (Török et al., 2014). Hence, HSP levels may increase without apparent protein denaturation or aggregation in the cell, such as when following a very mild temperature increase or in the presence of membrane-interfering compounds, such as Arimoclomol and Benzyl alcohol (de Marco et al., 2005; Saidi et al., 2005, 2009; Vigh et al., 2005).

Chemicals such as: Bimoclomol, Arimoclomol, Benzyl alcohol, HA (Hydroxyl amine) derivates, were found not to induce a “classical” elevation of the HSR. We and others have shown that these membrane fluidizers cause induction at non-inducing temperatures in plants and bacteria (Vígh et al., 1997; Balogh et al., 2005).

While these findings strengthen the hypothesis that membranes are “sensors” of environmental stress, through the modulation of microdomains (Escribá et al., 2008; Horváth et al., 2008), a number of investigators have explored “alternative” mechanisms by which a cell membrane receptor that is activated by external molecules can transmit signals that will eventually up-regulate the expressions of chaperones. A recent work in plants by Saidi et al. (Balogh et al., 2005) suggests that a mild temperature elevation or treatment with Hsp90 inhibitors induced the HSR via a transient influx of calcium through a channel in the plasma membrane. These experiments in plants suggest that early sensing of mild temperature occurs at the plasma membrane level and may be independent of the cytosolic protein unfolding machinery.

It was previously demonstrated that Hsp70 translocates into the plasma membrane after stress through the non-classical ER/Golgi transport pathway (Murakami et al., 2015; Multhoff et al., 1995). Murakami and others, suggested early in 1995 that lipid anchorage appears to be the most likely explanation for Hsp70 cell-surface localization (Murakami et al., 2015) and that the localization of surface Hsp70 is dependent on the cholesterol content of the membrane. It was also suggested that membrane Hsp70 may be associated with “lipid rafts” (Vega et al., 2008; Nimmervoll et al., 2015). Several researchers speculated that the possible mechanism behind this concept is that Hsp70 is transported to the plasma membrane by fusion of Hsp70–lipid vesicles with the plasma membrane. Recently, researchers have hypothesized that membrane Hsp70 may have a role in the endocytosis process in cancer (Nimmervoll et al., 2015); Elevated levels of membrane Hsp70 in melanoma cells through overexpression of the Hsp70 encoding gene induced clathrin- independent endocytosis. They further suggested that a fraction of the membrane Hsp70 that is localized in lipid rafts is oligomerized and clustered in the plasma membrane and that the Hsp70 membrane organization stimulated endocytosis (Nimmervoll et al., 2015).

The relevant pathways and mechanisms of the clathrin-independent endocytosis, relies on Calmodulin, Phospholipase D, PKA, PKC that are also known to be linked to calcium regulation. Small GTPases such as RhoA, Rac1, and ARF6 (Sandvig et al., 2011) are also involved. Among them, Rac1 was found to regulate actin polymerization and signals from integrins and growth factor receptors, therefore, also regulating cell transformation, tumor invasion, and metastasis (Gungor et al., 2014).

## Is there another pathway? is there a specific “heat sensing” receptor?

The direct heat-sensing or the perception of a non-thermal chemical stimulus by non-neuronal cells such as a HSR stimulus, resulting in the accumulation of HSPs, may be associated with changes in the membrane state, especially the organization of the membrane micro-domains, and the activation of a signal protein such as calmodulin (Balogh et al., 2005).

Transient Receptor Potential (TRP) channels are members of a large family of cationic channels that serve as major players in sensory physiology, such as transduction of chemicals, temperature, and mechanic stimuli (Török et al., 2014). TRP channels that are known to be gated by warm temperatures are TRPV1,2,3,4, and TRPM3. Among them the TRPV1, which is expressed in nociceptors and gated by heat, low pH Capsaicin (the active ingredient of the hot chili pepper), as well as by endogenous lipids, is known to be activated in inflammatory pain and hyperalgesia (Török et al., 2014).

Some studies have hypothesized that several TRP channels sense changes in temperatures via changes in lipid bilayer tension (Török et al., 2014) and that lipids have an important modulatory role in indirect TRPs gating. It has been suggested that thermodynamic changes in the lipid composition around these channels can regulate their gating as well (Multhoff et al., 1995).

There are several lipids that are known to modulate the TRP. However, up to now, the molecular mechanism modulating the temperature sensitivity of the TRP channels, is yet unknown. However, it has been demonstrated that the C terminus of the Transient Receptor Potential Vanilloid-1 (TRPV1) contains modulatory domains capable of binding calmodulin in the presence of bound Ca^2+^ ions (Jung et al., 2004).

TRPs are also sensitive to PIP2—which is an important feature of cell signaling due to its high abundance in the membrane and since it serves as a substrate for phospholipase C (PLC). However, the regulation of TRPV1 by PIP2 seems to be controversial: Although some studies have shown that activation of TRPV1 does not require PIP2, others have demonstrated that the addition of PIP2 can induce TRPV1 activation. It was also shown that oxidation of linoleic acid and LPA, another enzymatic product of phospholipids that is produced during inflammation and tissue injury, can activate TRPV1 as well. The cholesterol membrane content also plays a major role in the activity and sensitivity of several TRP channels such as TRPV1 and TRPC3.

**Figure 1:**
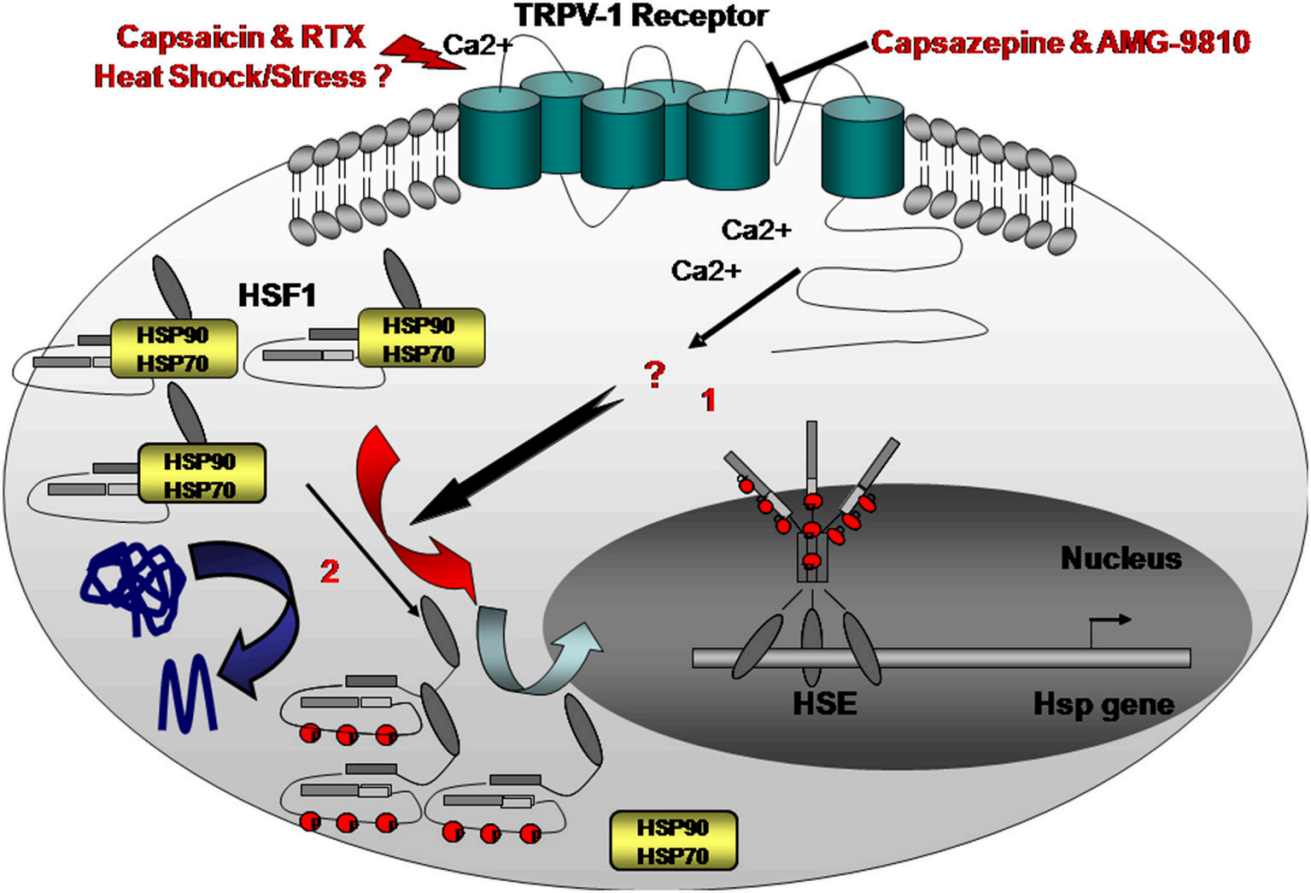
**Here we propose an alternative “heatsensing” model in which exposure to heat or treatment with capsaicin resulted in HSR activation, through TRPV1, by yet unknown interim cellular mediators (1) Capsazepine, a selective antagonist of TRPV1, abolishes the heat- or capsaicin-induced activation of the HSR and the consequent accumulation of Hsp70, 90, and 25 chaperones**. This model is an alternative to the current prevailing proposed mechanism suggesting that under stress, misfolded proteins recruit HSF1-interacting Hsps thereby allowing HSF1 activation (2).

Hence, ameliorations of lipid compositions and sphingolipid cholesterol signaling cascades during stress response or heat shock were found to be correlated with Hsp production (Török et al., 2014). Lipid membrane peroxidation was also found to activate HSF1. We recently found that there may be an alternative mechanism for activating the HSR through the membrane (Bromberg et al., 2013) (Figure 1). The use of Capsaicin or resiniferatoxin (RTX), both agonists of TRPV1, resulted in elevated levels of Hsp70, Hsp90, and Hsp25 in several epithelial cells in addition to a high abundance of nuclear HSF1 and higher HSF1 activity (Bromberg et al., 2013). In order to explore the existence of such a mechanism, EGTA, a calcium blocker, was added to the cells with or without heat shock treatment. Our findings were similar to those observed in plants; the addition of EGTA reduced heat-shock induced levels of Hsp70, Hsp90, Hsp25, as well as nuclear HSF1, suggesting that the calcium channels in mammals are involved in HSR regulation (Bromberg et al., 2013).

However, as mentioned earlier, the possible heat sensing membrane receptors as well as the downstream signaling pathways are still elusive. Several pathways and/or molecules are possible candidates. Activation of CaMKII, PI3-kinase, and ERK1/2 by Ca^2+^ influx has been reported to potentiate TRPV1 channel activity *in vivo* and *in-vitro* (Jung et al., 2004; Zhuang et al., 2004; Clapham, 2007). It is also known that phosphorylation of the TRPV1 channel by CaMKII can increase TRPV1 binding to Capsaicin and that ERK activation in rat DRG neurons occurs within 2 min after capsaicin application (Zhuang et al., 2004). Other downstream effectors of TRPV1 related to cellular cancer promoting pathways that may be associated with ERK and Src (Dai et al., 2002; Hwang et al., 2010).

## Modulation of membrane-associated heat shock receptor- the role of heat sensing cellular pathways in health and disease

It has been well described that membrane Hsp70 positive tumor cells are more resistant to irradiation than membrane Hsp70 negative cells (Murakami et al., 2015).

We have found that the addition of Capsaicin to breast cancer (MCF-7) cells, induced Hsp70 expression in some membrane areas (Bromberg et al., 2013).

We further demonstrated that *in-vivo*, TRPV1 was highly abundant in septic rats treated with an adenovirus overexpressing Hsp70 and that TRPV1 using co-immunoprecipitation, co-precipitated with Hsp70 from lung tissue of rats. These data may prove that during overexpression of Hsp70 or with the addition of Capsaicin, Hsp70 possibly co-localizes to the membrane and perhaps by that, regulates the HSR, an event that may occur in cancer cells as well (Bromberg et al., 2013).

Capsaicin, the agonists for the TRPV1, also resulted in elevated cytoplasmic levels of Hsp70, Hsp90, and Hsp25 in diverse cancerous epithelial cells, such as colon cancer cells (HT-29) and, as mentioned above, in MCF-7 cells.

Thus, we showed that the use of the membrane receptor blockers Capsazepine or AMG-9810, TRPV1 antagonists, and TRPV1 siRNA resulted in decreased heat shock proteins, Hsp70 and Hsp90 in cancer cells.

Therefore, we believe that a possible strategy to treat various cancers could utilize substances known to regulate the HSR through a membrane receptor capable of reducing the expression of heat shock proteins such as Capsazepine or AMG-132 and TRPV1 siRNA. Currently, we are exploring the consequences or the downstream pathways involved in this phenomenon (Ito et al., 2013).

## Targeting membrane and extracellular Hsp70 – an anti-Hsp70's directed cancer therapy

As described earlier, cancer cells or tumors frequently overexpress Hsp70 in the cytosol, but also present Hsp70 on their plasma membrane, as was found in our model as well (Vígh et al., 1997; Vega et al., 2008; Wiech et al., 2012). Membrane localization of Hsp70 in tumor cells is due to tumor-specific lipid composition of the membrane; Hsp70 co-localizes with the lipid raft glycolipidgloboyltriaosylceramide (Gb3) (Multhoff et al., 1997), and associates with phosphatidylserine (PS) which translocates from the inner to the outer plasma membrane through the activation of the ATP and Ca^2+^ after stress (Gehrmann et al., 2008).

The existence of membrane Hsp70 in tumor cells may also imply that there is a release of Hsp70 in lipid vesicles—termed exosomes (Schlegel and Williamson, 2001). Exosomes may fuse with the plasma membrane of target cells (Gastpar et al., 2005), transferring genetic material and signaling proteins, and thus may stimulate tumor metastasis (Campanella et al., 2014). Therefore, Hsps located on the surface of exosomes, secreted by normal stressed or tumor cells may have a possible central role in cell-to-cell communication (Gastpar et al., 2005). It was found that incubation of cells with exosomes, increased the phosphorylation of Hsp70 with ERK1/2, and was negatively regulated by caveolin-1 (Campanella et al., 2014). Extracellular Hsp70 may also be considered to act as “danger signals,” in modulating the cellular immune response (Svensson et al., 2013). Morimoto et al. (Moseley, 2000) have recently suggested a possible additional mechanism in which “Transcellular Factors” (TSF) or “danger signals” may be transferred through specific trans-cellular channels to adjacent cells, however, as a result of this chaperone trans-cellular pathway, the HSR is inhibited, or “shut-down” within the neighboring cells (Moseley, 2000). Serum levels of liposomal Hsp70 might also predict the presence of Hsp70 membrane-positive tumors. Studies have shown that Hsp70 serum levels are significantly higher in cancer patients compared to healthy individuals or patients with chronic inflammation. But it was also found that Hsp70 translocates into lysosomal membranes and by that stabilizes these membranes. Stabilized lysosomal membranes are protected against apoptotic stimuli, such as irradiation (Murakami et al., 2015).

Some cancer therapies such as thermo/radiotherapy could induce necrosis within the central zone. Thus, since elevated Heat shock protein levels can enhance tumor cell viability and increase resistance, the peripheral or transitional zone of sublethal hyperthermia might undergo a recovery from reversible injury, rendering this therapeutic approach inefficient.

However, rodent studies have indicated that irradiation was associated with a reduction in Hsp70 serum levels (Van Oosten-Hawle et al., 2013). Hence, Multhoff et al. proposed that serum Hsp70 levels could provide in the future a useful biomarker and a minimally invasive approach to predict the presence of tumors and to monitor the outcome of a therapy (Multhoff et al., 2015).

However, Hsp70 membrane expression, or exosomal Hsp70 on highly aggressive tumor cells could also activate NK cells (Multhoff et al., 2015):

Following irradiation, there is direct killing of cancer cells which is mediated by DNA damage, although this process also initiates effects that can stimulate the T and NK cell immune responses (Bayer et al., 2014). NK cells, which are related to the innate immune system, are also responsible for the first line of defense against malignantly transformed cells and tumors. It is known that NK cells can recognize membrane-bound Hsp70 on tumor cells (Wattenberg et al., 2014). Multhoff et al. (2015), have demonstrated that an incubation of NK cells with the Hsp70 protein or an Hsp70-derived peptide (TKD) with the addition of low doses of interleukin-2 (IL-2) for several days (Multhoff et al., 1998), can stimulate the proliferative and migration of NK cells against membrane- hsp70 positive tumor cells (Multhoff et al., 1998; Wattenberg et al., 2014).

These findings might have a significant future clinical relevance for irradiation therapy that could be improved by combining it with immunotherapy targeting membrane Hsp70.

## The importance of the plasma membrane in cancer therapy

The “cross talk” between the HSR, membranes, and intracellular pathways opens a new field that may help understand the mechanisms of missfolding diseases, such as aging and cancer. The plasma membrane fluidity state and its role in the pathological conditions of the cells, such as in cancer, aging, or stress response serves as the first guard against such stressors and connects between the extracellular and the intracellular signals. “Lipid therapy” as suggested by Vigh et al. in which a molecule affects membrane lipid re-organization maybe of therapeutic potential since such membrane fluidizers were found to enhance heat shock geneexpression in diseased cells without affecting healthy cells (Multhoff et al., 2001; Vigh et al., 2007).

Lipid therapy offers a possible strategy of regulating the fluidity of the membranes and modulating Hsps activities within the membrane. Hence, lipid therapy and antagonists of membrane-bound receptors, such as TRPV1, could allow us to modulate the heat sensing route of cancer cells, hence curtailing their pro-survival mechanisms.

## Author contributions

The authors ZB and YW are equally contributing authors to this current review. ZB and YW are co-collaborators for the last 15 years of joined research.

### Conflict of interest statement

The authors declare that the research was conducted in the absence of any commercial or financial relationships that could be construed as a potential conflict of interest.
